# Increasing Endocannabinoid Tone Alters Anxiety-Like and Stress Coping Behaviour in Female Rats Prenatally Exposed to Valproic Acid

**DOI:** 10.3390/molecules26123720

**Published:** 2021-06-18

**Authors:** Aoife M. Thornton, Rachel M. Humphrey, Daniel M. Kerr, David P. Finn, Michelle Roche

**Affiliations:** 1 Physiology, School of Medicine, National University of Ireland Galway, H91 W5P7 Galway, Ireland; aoifethorn@gmail.com (A.M.T.); r.humphrey1@nuigalway.ie (R.M.H.); 2 Galway Neuroscience Centre, National University of Ireland Galway, H91 W5P7 Galway, Ireland; david.finn@nuigalway.ie; 3 Centre for Pain Research, National University of Ireland Galway, H91 W5P7 Galway, Ireland; 4 Pharmacology and Therapeutics, School of Medicine, National University of Ireland Galway, H91 W5P7 Galway, Ireland; danny.kerr@nuigalway.ie

**Keywords:** autism, VPA, sex differences, social behaviour, anandamide, 2-AG, FAAH, MGL

## Abstract

Given the sex differences evident in the prevalence of autism, there is an increased awareness of the importance of including females in autism research to determine sexual dimorphism and sex-specific treatments. Cannabinoids and endocannabinoid modulators have been proposed as potential novel treatments for autism-related symptoms; however, few studies to date have examined if these pharmacological agents elicit sex-specific effects. The aim of the present study was to use the valproic acid (VPA) model of autism to compare the behavioural responses of male and female rats and examine the effects of increasing endocannabinoid tone on the behavioural responses of VPA-exposed female rats. These data revealed that VPA-exposed male, but not female, rats exhibit reduced social responding in the three-chamber and olfactory habituation/dishabituation (OHD) test during adolescence. In comparison, VPA-exposed female, but not male, adolescent rats exhibited anxiety-like behaviour in the elevated plus maze (EPM) and open field test (OFT). In VPA-exposed female rats, increasing 2-AG levels augmented anxiety-like behaviour in the EPM and OFT, while increasing AEA levels reduced stress coping behaviour in the swim stress test. These data highlight sexual dimorphic behaviours in the VPA model and indicate that enhancing endocannabinoid levels may exacerbate negative affective behaviour in VPA-exposed females. Thus, considerations should be paid to the possible sex-specific effects of cannabinoids for the treatment of symptoms associated with autism.

## 1. Introduction

Autism is a complex neurodevelopmental disorder characterised by two core symptoms—persistent deficits in social communication and interaction, and restricted, repetitive patterns of behaviours, interests or activities [1]. In addition to the core symptoms, autism is also associated with anxiety, irritability, GI disturbances and altered pain [2,3]. Autism is diagnosed three to four times more commonly in males than females [4,5]. However, increasing data suggests that the core symptoms of social deficits and restricted behaviours are more severe in males, which may account for the higher frequency of diagnosis [6,7]. Thus, examining these sex differences in animal models of autism is paramount to improving diagnosis and identifying appropriate sex-specific treatments. Animal models can provide important information in this regard. Valproic acid (VPA) is a widely prescribed and efficacious anti-epileptic and mood stabilizer. However, VPA is also a potent teratogen, associated with high incidences of birth defects and increased risk of developing autism in the offspring [8,9,10]. This clinical observation has led to the development of the VPA rodent model of autism, a widely used and clinically relevant rodent model displaying both behavioural and neurochemical changes observed in autism [9,11]. While much of the research conducted in this model has examined effects in males, only a few studies have directly compared effects in males vs. females. Deficits in social motivation are evident from a very early age in the nest-seeking test in VPA-exposed male [12,13,14,15,16] and female [16,17] rats. Deficits in social play behaviour, sociability and social novelty preference in male adolescent rats prenatally exposed to VPA has been a consistent finding in the model [14,15,18,19,20,21,22,23]. However, conflicting data have been reported for female rats, with some studies reporting social play deficits [12,24] and one with no change [25]. Similarly, several studies reported no deficits in the three-chamber test in VPA-exposed female rodents [12,26,27,28], while others have reported a deficit in sociability in VPA-exposed adolescent female rats [16,29] and once in adult rats [30]. Anxiety is the most common comorbid disorder associated with autism [31,32]. Anxiety-like behaviours have been reported in the VPA model in several paradigms including the elevated plus maze (EPM) and the open field test (OFT) [12,14,15,20,22,33], with the majority of these studies in male rodents. Increased anxiety-like behaviour has also been demonstrated in VPA-exposed female adult [24,34] and adolescent [24] rodents. Given that the majority of studies have examined male and female rodents separately, it is difficult to draw firm conclusions on the effect of sex on behavioural responding in the VPA model. 

The endocannabinoid system (ECS) is a key mediator and modulator of social and anxiety behaviour. Sex-specific effects of cannabinoid modulators have been reported in several conditions including anxiety and nociception [35,36,37]. Increasing clinical and preclinical evidence have implicated a key role for the ECS in the pathophysiology and treatment of symptoms associated with autism (for reviews, see [38,39,40]). Alterations in the ECS have been described in the VPA model including altered levels of the endocannabinoids anandamide (AEA) and 2-arachidonoylglycerol (2-AG) [41,42], altered mRNA and protein expression of components of the ECS [15,21,41,42] and changes in cannabinoid 1 (CB)_1_ receptor activation [12,15]. Only one paper to date has examined the effects in a sex-specific manner, demonstrating enhanced activation of CB_1_ in the prefrontal cortex (PFC) of VPA-exposed female, but not male, rats compared to saline-exposed counterparts [12]. Furthermore, VPA-exposed male, but not female, rats exhibited increased CB_1_ receptor activation in the dorsal striatum and reduced activation in the amygdala and hippocampus compared to saline-exposed rats [12,15]. Increasing AEA and 2-AG tone following the inhibition of the endocannabinoid-catabolising enzymes, fatty acid amide hydrolase (FAAH) and monoacylglycerol lipase (MGL), respectively, has proven effective at reversing several of the behavioural anomalies in VPA-exposed male rats including social impairments and anxiety- and stress-like behaviours [12,20,27,42]. Only two studies have examined the effects of enhancing AEA tone on behavioural responding of VPA-exposed females, demonstrating that FAAH inhibition attenuated the VPA-induced deficits in the DSI test during adolescence and repetitive-like behaviours during adulthood [12] but did not alter sociability in the three-chamber test, thermal nociception in the hotplate test (HPT) or repetitive behaviours during adolescence [27]. Despite the volume of studies that have investigated behavioural responding in the VPA model, there are very few that have directly examined a full range of behaviours in both male and female rats. Furthermore, while acute FAAH and MGL inhibition have been shown to reverse behavioural alterations in VPA-exposed male rats, there is a paucity of knowledge on whether this can be extended to VPA-exposed female rats. Therefore, the present study aimed to examine the effect of prenatal exposure to VPA on a range of behavioural changes in both male and female rats and the effect of acute FAAH and MGL inhibition on nociceptive and negative affective behaviours in VPA-exposed female rats.

## 2. Results

### 2.1. Prenatal VPA Exposure Induces Changes in Social Responding at Different Developmental Periods for Male and Female Rats 

**Figure 1:**
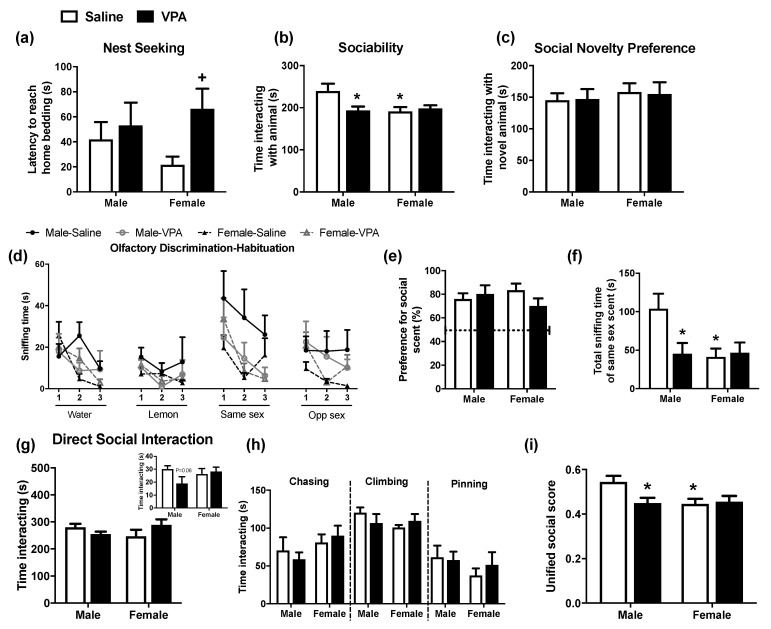
The effect of prenatal VPA exposure on social behaviour in male and female rats. (**a**) Latency to reach home bedding in the nest-seeking test at PND 13 (n = 7 to 10 per group). (**b**) Time interacting with animal in the sociability phase of the three-chamber test during adolescence (n = 10 to 11 per group). (**c**) Time interacting with novel animal in the social novelty preference phase of the three-chamber test during adolescence (n = 12 per group). (**d**) Time spent sniffing each scent (water, lemon, same sex, opposite sex), (**e**) discrimination index ((same sex)/(same sex + lemon) × 100) and (**f**) total time spent sniffing the same sex scent in the OHD test during adolescence (n = 7 to 12 per group). (**g**) Total social interaction during the 10 min DSI test and the first min of the trial and (**h**) chasing, climbing and pinning behaviour during the DSI test during adolescence (n = 6 per group). (**i**) Unified behavioural social score (n = 12 per group). Data expressed as mean + SEM. * *p* < 0.05 vs. saline-exposed males; ^+^ *p* < 0.05 vs. saline-exposed females.

Early social motivational behaviour was assessed in saline- and VPA-exposed pups using nest-seeking behavioural responding on PND 13. Analysis revealed a significant VPA effect (F_(1,30)_ = 5.29, *p* = 0.028) and post hoc analysis revealed that VPA-exposed female, but not male, pups had an increased latency to reach home bedding, indicative of reduced social motivation (Figure 1a). 

During adolescence, rats underwent three tests assessing social behaviour: the three-chamber test, OHD and DSI test. The three-chamber test is one of the most widely used tests for assessing sociability and social novelty preference in rodents [43]. In the sociability phase of the three-chamber test, analysis of time spent interacting with an animal revealed a significant VPA × sex interaction effect (F_(1,38)_ = 4.99, *p* = 0.031) [two-way ANOVA]. Post hoc analysis revealed that male VPA-exposed animals spent significantly less time interacting with the animal compared to saline-exposed counterparts, indicating a decrease in sociability behaviour in VPA-exposed male, but not female, rats in this paradigm (Figure 1b). Furthermore, saline-exposed female rats interacted less with the animal compared to the male counterparts, highlighting sex differences in social investigatory/motivational behaviour in this test. In the social novelty preference phase of the three-chamber test, there was no significant effect of prenatal VPA exposure or sex on the duration of time interacting with the novel animal (Figure 1c). Prenatal exposure to VPA did not alter the distance moved, rearing or grooming behaviour of male or female adolescent rats during the three-chamber test (data not shown).

As the social behaviour of rodents is highly dependent on olfactory cues, it is important to determine if animals exhibit normal olfactory function and are capable of detecting, recognising and distinguishing between odours including non-social and social scents. The OHD test assesses an animal’s capacity to habituate to an odour, which should be seen as a progressive decline in sniffing on repeated exposure to the olfactory stimulus (habituation) and subsequently the ability to recognise the introduction of a novel odour (dishabituation) [44], as observed in Figure 1d. Analysis of the social discrimination index revealed that all animals could distinguish between the social and the non-social scents and had a preference for the social odour (Figure 1e). Analysis of the total time spent sniffing the same sex odour, provides a further measure of social motivation, and revealed a significant effect of sex (F_(1,33)_ = 4.74, *p* = 0.037) and a VPA × sex interaction effect (F_(1,33)_ = 5.09, *p* = 0.031). Post hoc analysis revealed VPA-exposed male rats spent significantly less time sniffing the same sex odour than saline-exposed male counterparts, indicating a deficit in social motivation (Figure 1f). Furthermore, saline-exposed female rats spent significantly less time sniffing the same sex scent compared to male counterparts, an effect not altered by prenatal VPA exposure. Analysis also revealed no effect of VPA or sex on the total time spent sniffing water, lemon or opposite sex scents.

The DSI test is a widely used test to examine social motivation and reward in rodents [45]. Analysis of the total time engaged in social interactive behaviours during the 10 min DSI test did not reveal any significant VPA or sex effects; however, a trend was seen for a reduction in VPA-exposed male rats vs. saline counterparts in the first minute of the trial (Figure 1g). It has been reported that prenatal VPA exposure selectively reduces certain discrete social interactive behaviours [13,19,46]; however, analysis revealed no effect of VPA or sex on chasing, climbing or pinning behaviour in the test (Figure 1h). 

The unified social behavioural score generated a single score for the cohort of animals that underwent the three-chamber, OHD and DSI tests as adolescents. Analysis of this score revealed a significant VPA × sex interaction effect (F_(1,44)_ = 9.25, *p* = 0.04). Post hoc analysis revealed that VPA-exposed males and saline-exposed females had a lower overall social score than saline-exposed males (Figure 1i).

### 2.2. Prenatal VPA Exposure Induces Anxiety-Like Behaviour in Female, But Not Male, Adolescent Rats

**Figure 2:**
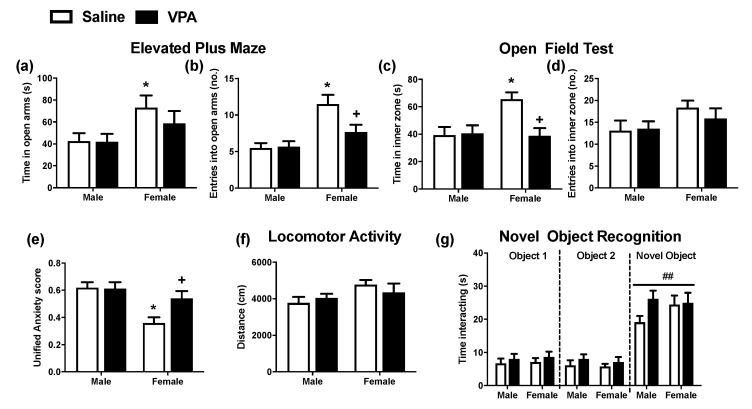
The effect of prenatal VPA exposure on anxiety-like behaviour and recognition memory in male and female adolescent rats. (**a**) Time in open arms and (**b**) number of entries into the open arms during the EPM (n = 12 per group). (**c**) Time in inner zone and (**d**) number of entries in the inner zone during the OFT (n = 12 per group). (**e**) Unified behavioural anxiety score (n = 12 per group). (**f**) Locomotor activity in open field arena (n = 12 per group) and (**g**) time interacting with familiar and novel objects during the NOR trial (n = 11 to 12 per group). Data expressed as mean + SEM. * *p* < 0.05 vs. saline-exposed males; ^+^ *p* < 0.05, vs. saline-exposed females. ^##^ *p* < 0.01 vs. corresponding group interacting with object 1 and 2.

Anxiety-like behaviour was assessed in the EPM and OFT during adolescence. In the EPM, analysis of the time spent in the open arms revealed a sex effect (F_(1,44)_ = 6.27, *p* = 0.016) and post hoc analysis demonstrated that female saline-, but not VPA-, exposed rats spent significantly more time in the open arms of the EPM when compared to the male counterparts (Figure 2a). Analysis of the number of entries into the open arms revealed an effect of sex (F_(1,44)_ = 17.55, *p* < 0.001) and VPA × sex interaction (F_(1,44)_ = 4.39, *p* = 0.042). Post hoc analysis demonstrated that female saline-exposed rats entered the open arms more frequently than male counterparts, an effect reduced by prenatal VPA exposure (Figure 2b).

In the OFT, analysis of the time spent in the inner zone of the arena revealed effects of VPA (F_(1,44)_ = 5.05, *p* = 0.03), sex (F_(1,44)_ = 4.66, *p* = 0.036), and VPA × sex interaction (F_(1,44)_ = 6.06, *p* = 0.018). Post hoc analysis revealed that female saline-exposed rats spent more time in the inner zone compared to male counterparts. Furthermore, VPA-exposed female rats spent significantly less time in the inner zone compared to the saline-exposed counterparts, indicating VPA-exposed female rats exhibited anxiety-like behaviour (Figure 2c). The number of entries into the inner zone was unaltered by prenatal VPA exposure or sex (Figure 2d), indicating that the effects are unlikely to be due to changes in locomotor activity. Analysis of rearing revealed a significant sex effect (F_(1,44)_ = 5.46, *p* = 0.024) and post hoc analysis found that female saline-exposed rats reared significantly more compared to male counterparts (male-saline: 26 + 5 s vs. female-saline: 44 + 7 s, *p* < 0.05), an effect not significantly different between male and female VPA-exposed animals. Grooming behaviour was not significantly altered by prenatal VPA exposure or sex. 

Analysis of the unified anxiety score revealed a significant effect of sex (F_(1,44)_ = 12.85, *p* = 0.001) and VPA × sex interaction (F_(1,44)_ = 4.09, *p* = 0.049). Post hoc analysis revealed that saline-exposed female rats had a significantly lower unified anxiety score compared to male counterparts, an effect not observed in VPA-exposed female rats (Figure 2e). 

### 2.3. Prenatal VPA Exposure Does Not Alter Locomotor Activity or Novel Object Recognition in Female or Male Adolescent Rats 

In order to determine if changes in social- or anxiety-related behaviour may be confounded by alterations in locomotor activity or recognition memory, these behaviours were also examined. Locomotor activity was not altered by sex or prenatal VPA exposure (Figure 2f). In the novel object recognition (NOR) test, analysis of the time spent interacting with the novel and two familiar objects revealed a significant effect of object (F_(2,129)_ = 61.58, *p* < 0.001) [three-way ANOVA]. Post hoc analysis revealed that all animals spent significantly more time interacting with the novel object vs. objects 1 and 2, thus indicating an intact recognition memory in all animals (Figure 2g). There was no significant effect of prenatal VPA exposure or sex on the time spent interacting with the novel object. 

### 2.4. Enhancing 2-AG and AEA Levels Alters Anxiety-Like and Stress Coping Behaviour in VPA-Exposed Female Rats, Respectively 

**Figure 3:**
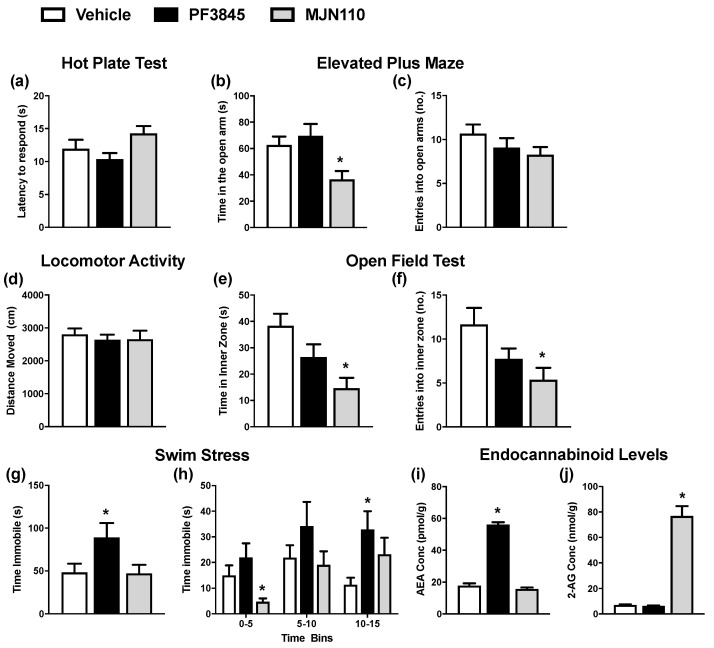
The effect of PF3845 (10 mg/kg) and MJN110 (5 mg/kg) on nociceptive and affective behaviour and endocannabinoid levels in VPA-exposed female rats during adolescence. (**a**) Latency to respond in the HPT. (**b**) Time in open arms and (**c**) number of entries into the open arms during the EPM. (**d**) Locomotor activity, (**e**) time in inner zone and (**f**) number of entries into the inner zone during the OFT. (**g**) Time spent immobile during the 15-min FST and (**h**) over-5-min time bins. (**i**) AEA and (**j**) 2-AG levels in the cortex. Data expressed as mean + SEM (n = 11 to 12 per group). * *p* < 0.05 vs. vehicle.

Previous data have indicated that enhancing AEA and 2-AG tone by inhibiting FAAH and MGL, respectively, attenuates social impairments in male rats prenatally exposed to VPA [12,15,20,27,42]. The data herein demonstrate that female rats prenatally exposed to VPA do not exhibit social impairments but rather anxiety-like behaviour. Thus, the effect of enhancing AEA and 2-AG tone following FAAH or MGL inhibition on anxiety-like behavioural responding in VPA-exposed female rats was examined. Animals were first exposed to the hot plate test (HPT) as the published data have shown that VPA-exposed female rats exhibit thermal hypoalgesia [24]. Neither PF3845 nor MJN110 altered the latency to lick the hind paws in the HPT (F_(2,33)_ = 2.87, *p* = 0.071) [one-way ANOVA] (Figure 3a).

Analysis of time spent in the open arms of the EPM revealed a significant drug treatment effect (F_(2,33)_ = 5.73, *p* = 0.007). Post hoc testing demonstrated that MJN110, but not PF3845, reduced the time spent in the open arms (Figure 3b). MJN110 or PF3845 did not alter the number of entries into the open arms (Figure 3c). In the OFT, locomotor activity was unaltered by drug treatment (Figure 3d). Analysis of the time and number of entries into the inner zone revealed significant drug treatment effects [time: (F_(2,32)_ = 6.74, *p* = 0.004); frequency: (F_(2,32)_ = 4.39, *p* = 0.021)]. Further post hoc analysis revealed that MJN110 reduces the time spent and number of entries of VPA-exposed females into the inner zone, thus inducing anxiogenic-like responses in these animals (Figure 3e,f).

In order to examine effects on stress coping behaviour, the animals were subsequently exposed to a swim stress and analysis of the time spent immobile revealed a significant drug treatment effect (F_(2,33)_ = 3.46, *p* = 0.043). Post hoc analysis revealed that PF3845 increased the time spent immobile in VPA-exposed rats over the 15 min trial (Figure 3g). Temporal analysis over 5 min time bins revealed a significant effect of drug treatment on the 0- to 5-min and 10- to 15-min time bins [0–5: (F_(2,30)_ = 6.73, *p* = 0.004); 10–15: (F_(2,33)_ = 3.52, *p* = 0.041)]. Post hoc testing showed MJN110 reduced the time spent immobile in the first 5 min, while PF3845 increased the immobility time in the 10- to 15-min time bin (Figure 3h).

Mass spectrometry analysis confirmed that systemic administration of PF3845 increased cortical AEA levels (F_(2,32)_ = 314.52, *p* < 0.001), while MJN110 increased cortical 2-AG levels (F_(2,32)_ = 158.89, *p* < 0.001) (Figure 3i,j).

## 3. Discussion

Autism is three to four times more prevalent in males than females [4,5]. Females with autism often demonstrate better social and communication skills and less severe patterns of repetitive and restrictive behaviours compared to male counterparts [6,7,47]. The present study demonstrated that systemic administration of VPA to pregnant Sprague–Dawley rats induces sex-specific autism-related behaviours in the offspring. Specifically, VPA-exposed adolescent male rats exhibited reduced social responding while VPA-exposed female rats exhibited a deficit in social motivation as neonates and an anxiety-like phenotype during adolescence. These effects were not confounded by changes in olfaction, locomotor activity or recognition memory. Furthermore, enhancing the levels of 2-AG enhances anxiety-related behaviour but increases stress coping behaviour in a time-dependent manner, while enhancing AEA levels reduces stress coping behaviour in VPA-exposed female rats. Overall, these data demonstrate sex-specific effects of prenatal VPA exposure on behavioural responding during the neonatal and adolescent period and indicate that enhancing endocannabinoid tone enhances anxiety and stress-like behaviour or stress in VPA-exposed female rats. Thus, unlike in males, where enhancing endocannabinoid tone elicits beneficial effects on autistic-like behaviours, in females, enhancing the 2-AG tone of this system may exacerbate anxiety-like behaviour. These data suggest sex-dependent effects of endocannabinoid modulators on behavioural symptoms associated with autism, which may have important implications for the use of such pharmacological modulators in this cohort of individuals. 

The present study demonstrated that VPA-exposed rats exhibit social deficits in multiple paradigms. For example, VPA-exposed female, but not male, neonatal pups (PND 13) exhibited reduced nest seeking behaviour. VPA-exposed female pups have previously been shown to exhibit a social motivation deficit in the nest-seeking test [17]. The lack of effects in VPA-exposed male neonates in this study may be accounted for by different strains (Sprague–Dawley vs. Wistar) and different ages at testing (PND 13 vs. PND 9) [14,15,16]. Thus, we cannot rule out that VPA-exposed male neonatal pups may have exhibited a deficit in social motivation at a time other than that tested in the current study. During adolescence, VPA-exposed male rats exhibited a deficit in sociability/social motivation in the three-chamber test. This is in line with several other studies, including those from our own laboratory, showing VPA induced a decrease in sociability in male adolescent rats [19,27,41]. Prenatal VPA exposure did not alter social novelty preference responding, similar to two studies that demonstrated that VPA-exposed male rats exhibited a deficit in sociability but a normal social novelty preference [29,48]. However, it should be noted that other studies have shown that prenatal exposure to VPA induces a deficit in social novelty preference/social cognition in male adolescent rats [22,23,49], indicating that the effects on social novelty preference are variable and reflect subtle changes in experimental design. Data from the OHD test provided further support for social impairments in VPA-exposed male rats. These data mirror the three-chamber sociability data as VPA-exposed male rats spent less time sniffing the novel social scent (same sex) compared to saline counterparts. This is similar to studies which demonstrated that VPA-exposed male mice spent less time sniffing the social odour during its first presentation [50] and rats spent less time sniffing urine compared to the control group [46,51]. Taken together, VPA results in sexual dimorphisms in the motivation to socially interact with the same sex, an effect dependent on olfaction rather than the presence of the animal. These data confirm that VPA does not alter olfaction as all animals are capable of distinguishing novel odours. In line with the numerous published reports [12,14,15], data from our laboratory have confirmed that when examined as the first behavioural test, male rats prenatally exposed to VPA (500 mg/kg) spent less time engaged in DSI (unpublished). However, in this current study, no significant differences were evident in the DSI test, although a trend for a reduction was noted in the first minute of the test in VPA-exposed male rats. This was surprising but may be explained by the battery of tests the animals underwent prior to the DSI, the repeated handling, and that they were singly housed for 11 days, when maximal pinning behaviour has been reported after isolation for 24 h [52]. When data from the three-chamber, OHD and DSI tests are combined to give a single unified social score, a significant reduction in sociability is evident in VPA-exposed male, but not female, adolescent rats, further supporting sex-specific effects of VPA on social responding. The data herein also confirmed that the VPA-induced social impairments are independent of changes in locomotor activity, olfaction or short-term recognition memory and thus VPA appears to selectively alter the social network of male rodents [12,16,53].

VPA-exposed female Wistar rats have been reported to demonstrate social deficits in an age-dependent manner, exhibiting normal social behaviour as juveniles in the nest-seeking test and as adults in the three-chamber test, but reduced social play as adolescents in the DSI test [12]. In comparison, the data in the current study demonstrate that prenatal exposure to VPA did not induce social deficits in female adolescent rats in the three-chamber, OHD or DSI test. Similarly, the published studies from our own laboratory and others have reported no change in sociability in VPA-exposed female rats during the three-chamber [12,27,28] and DSI test [25]. It should also be noted that female rats engaged in less social motivational/investigative behaviour compared to the male counterparts, a finding in line with previous data [54]. Thus, due to the low level of social interactive behaviour of female rats in the three-chamber paradigm, it may not be possible to reveal further decreases in social responding in female rats under the current experimental conditions. Similar effects were observed in the OHD test as no differences were detected between saline- and VPA-exposed female rats but saline-exposed female rats had a lower duration of time spent sniffing compared to male counterparts. A recent study reported VPA-exposed female adolescent rats exhibited normal social play behaviour but reduced USVs during the DSI test [25]. Thus, although female VPA-exposed rats may not exhibit a social motivational deficit, they may exhibit a social communication deficit that could be revealed by assessing USVs.

In contrast to the lack of effects on social responding, VPA-exposed female adolescent rats exhibited anxiety-like behaviour in the EPM and OFT. This is similar to previous studies that reported an anxiety-like phenotype in VPA-exposed female rodents during adolescence [24] and adulthood [24,34,55]. It is somewhat surprising that VPA-exposed male rats did not also exhibit an anxiety-related phenotype given the wealth of previous studies demonstrating anxiety-related behaviour [12,15,20,22,23,24,49,56]. Differences between studies may be accounted for by different species and strains used, testing in a single instance or as part of a battery of tests, or developmental age. However, under the current experimental conditions, prenatal VPA exposure is associated with an anxiety-related phenotype in female, but not male, adolescent rats.

**Figure 4:**
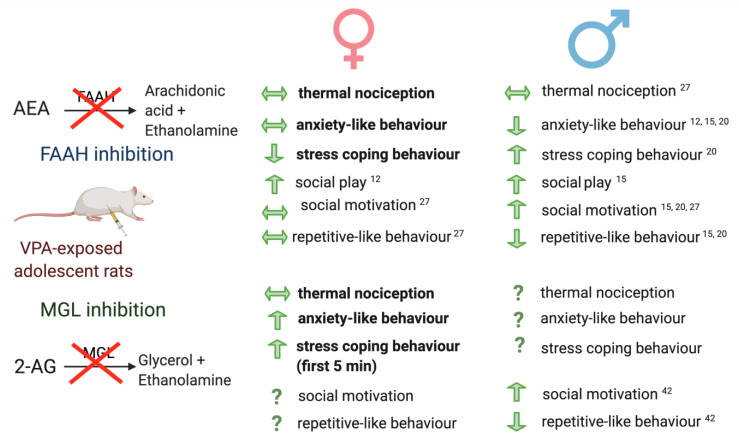
Schematic depicting the effects of FAAH and MGL inhibition on anxiety-like behaviour in VPA-exposed female and male rats. Bold highlights the data depicted in the current study. References provided for data cited in published literature.

Acute FAAH inhibition reverses social impairments, cognitive deficits, repetitive- and anxiety-like behaviours and increases stress coping behaviours in VPA-exposed male adolescent rats [12,15,20,27], while acute MGL inhibition rescues social deficits and repetitive-like behaviours [42] (Figure 4). Only two studies have examined the effect of FAAH inhibitors in VPA-exposed female rats, which was shown to reverse the decreased response to play solicitation in the DSI test during adolescence and repetitive-like behaviours during adulthood [12] but did not alter nociceptive, repetitive-like or social behaviour in the three-chamber test during adolescence [27]. To our knowledge, this is the first study to examine enhancing endocannabinoid tone on negative affective behaviours in VPA-exposed female rats. These data show that enhancing AEA levels did not alter anxiety-like behaviour in the EPM or OFT but reduced the stress coping ability in the FST in VPA-exposed female rats. This is in contrast to the published literature which demonstrated that acute FAAH inhibition reduced anxiety-like behaviour in the EPM and increased stress coping behaviour in the FST in VPA-exposed male adolescent rats [15,20] (Figure 4). Discrepancies are likely due to differences in endocannabinoid tone and signalling between VPA-exposed male and female rats. Sex differences have been reported in the activation of CB_1_ receptors in the PFC, dorsal striatum, amygdala and hippocampus which may contribute to these differential responses [12]. The aforementioned studies, which examined FAAH inhibition in VPA-exposed male rats, did not measure AEA levels; it is possible that the increases in the female rats were greater than that in male rats and therefore resulted in the activation of TPRV1 instead of CB_1_, which would lead to anxiogenic, not anxiolytic, effects [57]. It is important to note that the increase in immobility in the swim stress test is unlikely to be due to locomotor suppression as neither drug altered the distance moved. Thus, the effects are specific for stress coping behaviour.

In contrast to the lack of effect of FAAH inhibition, the present data demonstrate that increasing 2-AG tone augments anxiety-like behaviour of VPA-exposed females in the EPM and OFT. To our knowledge, this is the first study to examine the effect of MGL inhibition on these behaviours in VPA-exposed rodents. MGL inhibitors have been demonstrated to elicit anxiolytic effects [58,59]; however, these effects are only evident when the animal is subjected to stress or under high aversive conditions and the majority of published work was carried out in male adult rodents. Interestingly, MGL inhibition increased stress coping behaviour in the FST during the first 5-min time bin. The mechanism underlying these time-dependent effects remain to be determined. It should also be noted that neither FAAH nor MGL inhibition altered thermal nociceptive responding in VPA-exposed female rats. While this is in line with previous studies demonstrating a lack of effect of FAAH inhibition on hypoalgesia on the hotplate test in VPA-exposed female (and male) rats [27], to our knowledge, this is the first study to examine the effects of MGL on thermal nociception in VPA-exposed rodents (Figure 4). Taken together, FAAH and MGL inhibitors attenuate autism-related symptoms in VPA-exposed male rats; however, they may exacerbate negative affective behaviours in VPA-exposed female rats. 

In conclusion, these data demonstrate that VPA induces social impairments in a sex- and age-specific manner. VPA-exposed male, but not female, adolescent rats exhibit social deficits while VPA-exposed female, but not male, adolescent rats exhibit anxiety-like behaviour. Increasing the AEA and 2-AG levels enhanced stress coping behaviour and anxiety-like behaviour, respectively, in VPA-exposed female rats, indicating that while these drugs may be beneficial in VPA-exposed male rats, they do not attenuate negative affective behaviours in VPA-exposed female rats.

## 4. Materials and Methods 

### 4.1. Animals and Prenatal Administration of Valproic Acid

Male and female Sprague–Dawley rats (200 to 340 g; Charles River Laboratories, Harlow, UK) arrived into the facility, were group-housed and allowed one week of acclimatization prior to being paired for mating. The presence of spermatozoa in vaginal smears as determined by microscopy indicated the first day of gestation (GD 0.5), after which pregnant female rats were singly housed. Housing conditions were maintained at constant temperature (20 to 24 °C) and humidity (40 to 50%) under standard lighting conditions (12:12 h light–dark cycle, lights on from 07.00 to 19.00 h). Food and water were available ad libitum. On GD 12.5 female rats received a single subcutaneous (s.c.) injection of sodium valproate (VPA) (Sigma, Dublin, Ireland) (500 mg/kg) or sterile saline (0.89% NaCl) vehicle in an injection volume of 2 mL/kg. Females raised their own litters and pups were weaned on PND 21. Reproductive data (number of dams, gestational weight gain and length) and offspring developmental data (number of pups born and weaned, pup’s weight, eye opening and number of tail kinks in VPA-exposed litters) were recorded and revealed no significant difference between saline- and VPA-treated dams or -exposed litters (see Supplementary Table S1). VPA-exposed litters exhibit tail kinks in 50% of offspring (males: 54%; females: 46%). One to two pups per litter were used for the experiments. Experimental procedures were carried out under approval from the Animal Care and Research Ethics Committee at NUI Galway, under license from the Health Products Regulatory Authority and in compliance with the ARRIVE guidelines and the European Communities Council directive 2010/63/EU.

### 4.2. Experimental Design

**Figure 5:**
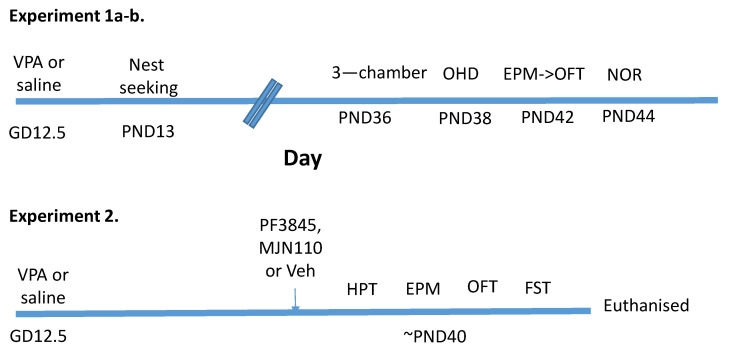
Schematic of the experimental design for the behavioural characterisation of male and female rats prenatally exposed to saline or VPA (experiment 1a–b) and examining the effect of enhancing AEA and 2-AG tone on nociceptive and affective behaviour in VPA-exposed female rats (experiment 2).

Experiment 1: Behavioural characterisation of male and female rats prenatally exposed to VPA. One cohort of saline- and VPA-exposed male and female pups underwent the nest-seeking test at PND 13 (experiment 1a), while another cohort underwent a battery of behavioural tests during adolescence beginning on PND 36–39 and continuing for 10 days (experiment 1b) (Figure 5). The sequence of testing remained constant. Animals were singly housed 24 h before the three-chamber test; two days later, they underwent the OHD test; four days later, they underwent the EPM, immediately followed by the OFT. The following day, they were habituated to the open field arena and the locomotor activity was examined, and 24 h later, they underwent the NOR test. DSI was carried out two days following NOR in pairs of the same treatment group.

Experiment 2: The effect of FAAH or MGL inhibition on nociceptive and affective responding in females rats prenatally exposed to VPA was examined. VPA-exposed female adolescent rats (PND 33–43) were randomly assigned to one of three treatment groups: vehicle (n = 12), the FAAH inhibitor PF3845 (n = 12) or MGL inhibitor MJN110 (n = 12). Animals were singly housed 24 h before testing. The FAAH inhibitor PF3845 (*N*-3-Pyridinyl-4-[[3-[[5-(trifluoromethyl)-2-pyridinyl]oxy]phenyl]methyl]-1-piperidinecarboxamide) (10 mg/kg; NIMH drug synthesis programme US), the MGL inhibitor MJN110 (4-[*bis*(4-chlorophenyl)methyl]-1-piperazinecarboxylic acid, 2,5-dioxo-1-pyrrolidinyl ester) (5 mg/kg; gifted by Dr Ben Cravatt, Scripps Institute, La Jolla, CA, USA) or vehicle (ethanol: cremophor: saline; 1:1:18) were administered i.p. in an injection volume of 2 mL/kg and animals were returned to their home cage for 2 h. The dose and time of drugs were chosen on the basis of previous published work [27,60,61,62]. The animals underwent behavioural testing in the following order: HPT, EPM, OFT and the FST. The animals were returned to their home cage for 10 min, after which time they were euthanised by decapitation, the brain removed and snap frozen on dry ice and stored at −80 °C until analysis for endocannabinoid levels (Figure 5).

### 4.3. Behaviour

All behavioural testing was carried out during the light phase between 8.00 and 16.00 h. All behavioural analysis was carried out by an experimenter blinded to group identity. 

#### 4.3.1. Nest Seeking

On PND 13, a male and female pup from each litter was randomly selected and separated from their dam and placed into a clean home cage which was lightly covered with fresh bedding and placed on a heating pad. The test was carried out as previously described, with slight modifications [13]. One corner of the home cage contained dirty bedding from their home cage and the other corner contained clean bedding. The pup was placed in the centre of the far side of the cage and the latency to reach the home bedding was recorded. A cut-off point of 120 s was set. 

#### 4.3.2. Three-Chamber Test

The animal was placed into a novel arena composed of three communicating compartments separated by Perspex walls with central openings allowing access to all chambers and behavioural testing was carried out as previously described [19,27,41,63]. Following a 10-min habituation to the test arena, the animals were briefly confined to the central compartment while an unfamiliar rat (animal) was introduced to one chamber under a small wire cage and an empty wire cage (object) was introduced to the other chamber (sociability phase). The test animal was allowed to explore the arena for 10 min. Subsequently, the test animal was briefly confined again, a novel unfamiliar rat was introduced under the empty wire cage and the animal was allowed to explore for a further 10 min (social novelty preference phase). Behaviour was recorded and evaluated with the aid of EthoVision XT 11.5 software (Noldus, Wageningen, The Netherlands). The behaviours assessed included the time interacting with the animal in the sociability phase, time interacting with the novel animal in the social novelty preference phase, distance moved, rearing and grooming. 

#### 4.3.3. Olfactory Habituation/Dishabituation Test

The animal was first acclimatised to the home cage for 5 min with a dry cotton bud in the cage and the test was carried out as previously described [50]. Cotton buds which had been soaked overnight in a scent were then introduced into the home cage and the animal’s approach was recorded over a 2-min period. Subsequently, the cotton bud was changed quickly so that each odour was presented three times. Four scents were used in this test—water, lemon, same sex bedding, opposite sex bedding. Time spent (s) sniffing the cotton bud was scored with the aid of EthoVision XT 11.5 to evaluate olfactory habituation and dishabituation. A discrimination index was calculated as (time sniffing same sex/(time sniffing same sex + time sniffing lemon) × 100).

#### 4.3.4. Direct Social Interaction

Two animals of the same treatment group were placed in a clean home cage under low lighting conditions (20 lux) and their behaviour was recorded for 10 min. The duration of time (s) spent in social interaction (chasing, climbing, pinning) was analysed with the aid of EthoVision XT 11.5 software.

#### 4.3.5. Elevated Plus Maze

The animal was placed in the centre of the EPM facing an open arm as previously described [41,64]. The animal was allowed to explore the open (90 lux) and closed (30 lux) arms freely for 5 min. The time spent in open arms (s) and the number of entries into open arms (no.) were recorded and analysed with the aid of EthoVision XT 11.5 software.

#### 4.3.6. Open Field Test

The animal was placed in the centre of the brightly lit (200 to 230 lux) novel open field arena (diameter 75 cm), as previously described [41,64]. The animal was allowed to explore freely, and distance moved (cm), time spent (s) in the inner zone (diameter 50 cm) of the arena and the number of entries into the inner zone were analysed with the aid of EthoVision XT 11.5 software.

#### 4.3.7. Novel Object Recognition

The animal was placed in the centre of the open field under dimmer lighting conditions (30 lux) and locomotor activity was recorded for 10 min. On the subsequent day, the animal was placed into the same arena, which now contained three identical objects. The animal was allowed to explore for 3 min and then returned to its home cage for 2 min. This process was repeated, and during the second 2 min break, one of the objects was switched to a novel object that was different in size and shape to the original object, and the animal was placed back into the arena for a further 3 min. Time spent (s) exploring each of the three objects during the trials were recorded and analysed with the aid of EthoVision XT 11.5 software.

#### 4.3.8. Hot Plate Test

The animal was placed on the hot plate and thermal nociception was measured as previously described [27,41]. The time elapsed (i.e., latency to respond (s)) between the placement of the animal on the surface of the hot plate and when the animal first licked either of its hind paws was recorded, with a cut-off time of 40 s to avoid tissue damage.

#### 4.3.9. Forced Swim Stress

The animal was placed in a glass cylinder (height: 45 cm, diameter: 20 cm) containing 30 cm of 22 to 23 °C water under 30 lux lighting conditions for a 15 min period. Duration of time spent (s) immobile was recorded and analysed with the aid of EthoVision XT 11.5 software.

### 4.4. Unified Behavioural Scoring

The unified behavioural score was introduced with the aim of maximising the use of all the data generated while minimizing statistical error [65]. It is particularly useful when animals have undergone a battery of behavioural testing. In this current study, we calculated two scores—social and anxiety. The method to calculate the unified behavioural score was applied as previously described [65]. 

A social score was calculated from behaviour in the sociability and social novelty preference phases of the three-chamber test, the OHD test and the DSI; an anxiety score was calculated from behaviour in the EPM and OFT. Outcome measures for each test were normalised by dividing the individual measure by the maximum measure in the study group to obtain a measure score between 0 and 1. The outcomes were assigned as either positive or negative factors such that a positive factor increases as sociability/anxiety increases (e.g., time interacting with animal) and a negative factor decreases as sociability/anxiety decreases (e.g., time interacting with object). Negative factors were subsequently inverted, and individual test scores were calculated as the average of all outcome measures associated with that test. The scores for each test were averaged to generate a single unified score for each behavioural trait for each rat. 

### 4.5. Quantification of Endocannabinoid Concentrations Using Liquid Chromatography–Tandem Mass Spectrometry

The quantitation of endocannabinoids (AEA and 2-AG) in the brain tissue was carried out as previously described [61]. In brief, prefrontal cortical samples were sonicated in 400 μL 100% acetonitrile containing deuterated internal standards (0.014 nmol AEA-d8, 0.264 nmol 2-AG-d8), centrifuged at 14,000× *g* for 15 min and the supernatant was collected. Samples were then separated on a Zorbax^®^ C18 column (50 × 2.1 mm internal diameter, 1.8 µm particle size) by reversed-phase gradient elution, initially with a mobile phase of 65% acetonitrile with 0.1% formic acid, which was ramped linearly up to 100% acetonitrile with 0.1% formic acid over 7 min and held at this for a further 5 min (flow rate 200 µL/min). Under these conditions, AEA and 2-AG were eluted at the following retention times: 3.5 and 4.7 min, respectively. Analyte detection was carried out in electrospray-positive ionization and multiple reaction monitoring mode on an Agilent 1100 HPLC system coupled to a triple quadrupole 6460 mass spectrometer (Agilent Technologies Ltd., Cork, Ireland). Quantification of each analyte was performed using Masshunter Quantitative Analysis Software (Aligent Technologies, Cheshire, UK). The limits of detection for analyte quantifications were as follows; 1.3 pmol/g and 12.1 pmol/g for AEA and 2-AG, respectively.

### 4.6. Statistical Analysis

SPSS (IBM, New York, NY, USA) statistical package was used to analyse all the data. Normality and homogeneity of variance were assessed using Shapiro–Wilk and Levene’s test, respectively. Normality and homogeneity were assumed when *p* > 0.05. Where appropriate, when comparing the means of two unrelated groups, parametric data were analysed using Student’s unpaired *t*-test. One-way ANOVA was used to compare the means of more than two groups while assessing one factor (i.e., drug treatment), two-way ANOVA was used while assessing two factors (i.e., VPA and sex) and three-way was employed while assessing three factors (i.e., VPA, sex and object). Post hoc analysis was performed using Duncan’s post hoc test where appropriate. Data were considered significant when *p* < 0.05. All graphs representing data were made using GraphPad Prism 8.0 and data were expressed as mean + SEM.

## Supplementary Materials

The following are available online, Table S1: The effect of prenatal VPA exposure on gestational and developmental milestones.

## Abbreviations

2-AG: 2-Arachidonoylglycerol; AEA: anandamide; CB_1_: cannabinoid receptor 1; DSI: direct social interaction; EPM: elevated plus maze; FAAH: fatty acid amide hydrolase; ECS: endocannabinoid system; fatty acid amide hydrolase; FST: forced swim test; GD: gestational day; HPT: hot plate test; MGL: monoacylglycerol lipase; NOR: novel object recognition; OFT: open field test; PND: postnatal day; PFC: prefrontal cortex; VPA: valproic acid.
