# Supplementary material for: Increasing Endocannabinoid Tone Alters Anxiety-Like and Stress Coping Behaviour in Female Rats Prenatally Exposed to Valproic Acid
Source: Molecules. 2021 Jun 18;26(12):3720. doi: 10.3390/molecules26123720 (PMC8233839; doi:10.3390/molecules26123720)
Supplement: Supplementary file 1 [file molecules-26-03720-s001.zip › molecules-1264403-supplementary.pdf]

Table S1. The effect of prenatal VPA exposure on gestational and developmental milestones. Data expressed as mean + SEM. a=GD0-GD17.

	Dams that gave birth (no.)	Dam weight gain (%) [a]	Pregnancy length (days)	Pups born (no.)	Pups weaned (no.)	Pup weights (g)			Eye opening (%)	
						PND7	PND14	PND21	PND13	PND14
**Saline**	12	40.1+2.1	21.2+0.2	10.8+ 0.95	9.8+ 0.8	14.6+1	31.5+1.7	52.7+2.3	18.8+7.8	47.7+14
**VPA**	14	43+2.4	21.2+0.3	10.43+ 0.98	8.6+ 1.1	15.7+0.8	32.3+0.9	52.9+1.7	26.1+12.3	73.3+7.9
